# A Model for Strengthening Mentors: Frames and Practices

**DOI:** 10.3390/ijerph18126465

**Published:** 2021-06-15

**Authors:** Stacy Blake-Beard, Mary Shapiro, Cynthia Ingols

**Affiliations:** 1 Tuck School of Business, Dartmouth College, Hanover, NH 30755, USA; 2 School of Business, Simmons University, Boston, MA 02115, USA; mary.shapiro@simmons.edu; 3 Institute for Inclusive Leadership, Simmons University, Boston, MA 02155, USA; cynthia.ingols@simmons.edu

**Keywords:** mentoring, leadership, development

## Abstract

The extensive body of literature on mentoring has largely ignored the developmental needs of mentors themselves. This conceptual and practice-oriented paper asks mentors and others to consider the needs of mentors who may or may not arrive ready to deal with the challenges of being effective mentors. The authors ask: how should mentors think about their own growth and development? Drawing on a broad spectrum of academic literatures, three frames are proposed for guiding mentors’ thinking about themselves and four practices to spur their continuous improvement. The three frames are a simultaneous dual focus on people and tasks as mentors exercise leadership; an inclusive mind-set that works across the multidimensionality of identities in others and themselves; and a keen sense of the threats and rewards of managing the perceptions of others. We recommend the use of four practices for self-examination: engage in structured self-reflection; participate in standardized assessments to see others and one’s self differently; build peer support among colleagues; and ask for feedback in concrete terms. We conclude by offering the benefits and challenges as mentors engage in the difficult work of acquiring in-depth self-awareness.

## 1. Introduction

Mentoring is a topic that garners great attention in both the research literature and the popular press as it addresses several critical issues. Organizations struggle to recruit and retain a qualified workforce [1,2]. The mentor–mentee relationship remains a central strategy of developing, supporting and retaining an organization’s employees while developing tomorrow’s leaders [3]. Employees’ loyalty to their organizations is enhanced if they perceive a culture that promotes their careers, compensates them fairly, and is psychologically safe [4]. In addition, leaders need the critical ability to leverage differences across an increasingly diverse workforce in an inclusive manner [5].

Studies and testimonials articulate the benefits that mentees receive as an outcome of mentoring relationships [6,7,8] and for the organization itself [9,10]. Benefits to mentees include having access to a source of encouragement and support [11,12]; learning practice-oriented skills; dealing with emotional and psychological distress; and navigating the job market [13]. Mentors are critical sources of wisdom and guidance for mentees who are following them in their organizational journey.

Given the strategic role that mentors play, it is important to have a strong understanding of what supports mentors’ ability to perform at their best. However, research on mentors’ experiences is scarce [14,15,16]. The research on mentors speaks to their experiences in the context of the mentoring relationship, but, what do they gain from the relationship as mentors?

In this paper, we focus on mentors’ own need for growth and development, as well as what organizations can do to address their needs. We start by sharing a contemporary definition of mentoring as well as the traditionally discussed benefits of mentoring to mentees and organizations. Doing so illuminates that the experience of mentors is often a missing piece in the puzzle of training, development, and support customarily seen in organizations. In the Methodological Underpinnings, we explain our process of developing the model. In our discussion of each frame and practice we draw from the literature to support and illuminate each concept further (For an extensive view of the mentoring literature, see Murrell and Blake-Beard’s edited volume—*Mentoring Diverse Leader: Creating Change for People, Processes, and Paradigms*. Routledge: New York, NY, USA, 2017). Finally, we close with the benefits and challenges to both mentors and their organizations in working towards generativity and suggest research that could examine the impact of that work.

## 2. Mentoring Definitions and Characteristics

Mentoring in the workplace is best understood as a process through which an individual (mentee) develops a relationship with a professional colleague (mentor) that typically contributes to the development and career growth of both [17]. Mentors are generally defined as individuals with advanced experiences and knowledge who commit to providing upward mobility and career support to their mentees. Contemporary definitions build on Kram’s germinal research— “mentoring is a long-term relationship that meets a developmental need, helps develop full potential, and benefits all partners, mentor, mentee and the organization” [18]. An examination of the chronological evolution of the process “shows that mentoring is widened from focus on skills development to include personal development by a more experienced person” [19].

Mentoring relationships are enacted through a set of behaviors that cover the spectrum of career to psychosocial support behaviors. For the career aspects of mentoring, we see the mentor providing coaching, exposure and visibility, sponsorship, access to challenging assignments, and protection. The psychosocial dimensions that are encompassed include role modeling, counseling, friendship, and acceptance [17] (see Table 1). Kram’s germinal research [12] also identified four phases through which mentoring relationships evolve. The mentor and mentee move from initiation (stage 1) to cultivation (stage 2): this progression represents the movement from the beginning of the relationship to an optimal level of career and psychosocial support. The third stage is separation: during this phase, the mentor and the mentee have grown apart from one another for a variety of physical and/or psychological reasons. During the fourth stage, redefinition, mentors and mentees may find a new equilibrium for their relationship, taking their connection to a different plane. At other times, redefinition marks the conclusion of the relationship.

Throughout these traditional definitions of functions and phases of mentoring, there is a focus on the mentee. The concepts were created with the mentor being in relation to the mentee, but one does not learn what will benefit and support mentors in their development. Erickson (1950)’s theory of development [20], which has been applied to mentoring, may shed some light on the needs of mentors. In fact, Kram’s model draws from Erickson’s theory, focusing on the seventh of eight stages of human beings: the question of generativity versus stagnation. Generativity represents a distinct desire to make an impact on the next generation. Stagnation represents the feeling that there has been a failure in finding ways to contribute [21]. Leaders who experience generativity feel concern for the next generation, and wish to make an impact on the world that will outlast them. Leaders experiencing stagnation may be disconnected from their organizations, communities, or society. We offer developmental activities that mentors may use to support their generativity and their roles as leaders.

## 3. Methodological Underpinnings

This literature review is grounded in the authors’ broad and thorough knowledge of the collected works on mentoring, leadership, and social identities. In addition, we acknowledge being mentees, serving as mentors for years, and establishing and teaching in mentoring and leadership programs for decades. Indeed, the authors believe: research has informed our practice, and our practice has informed our research. Both have informed this paper.

The literature on mentoring is extensive. Equally importantly, it edges into other bodies of literature, such as the leadership and diversity and inclusion fields. The authors pulled from these multiple bodies of knowledge and then distilled them to the areas that are discussed in this paper. This reflects our views on what we have found most relevant from research projects—both our own and others—and in the myriad books and articles on the topics.

The authors’ academic research has been blended with field work as faculty, coaches, and consultants. We have honed our awareness and expertise in what works—and what does not—to develop mentors and leaders. Our three frames reflect what we have most consistently heard executives struggle with, and the four practices are how we have most frequently responded to bring clarity to practitioners’ thinking. The proposed conceptual model both augments the existing literature and captures our best practices from our field work.

Individually and collectively, we developed executive and managerial educational programs and these experiences highlighted various propensities noted in the literature. We saw, for example, that executives often focused on either task or people rather than both, when they completed standardized assessments and team exercises. With such insights, we coached and mentored executives to expand their visions of particular situations. In other programs, we discussed peer mentoring, how it works, and its benefits; we then assigned people to triads where they practiced mentoring and receiving feedback on their observations. In short, our field work from multiple programs, consultancies, and coaching sessions—grounded in our deep knowledge of the mentoring literature—underpins the conceptual model in how to develop mentors.

## 4. Frames and Practices to Strengthen Mentors

**Figure 1:**
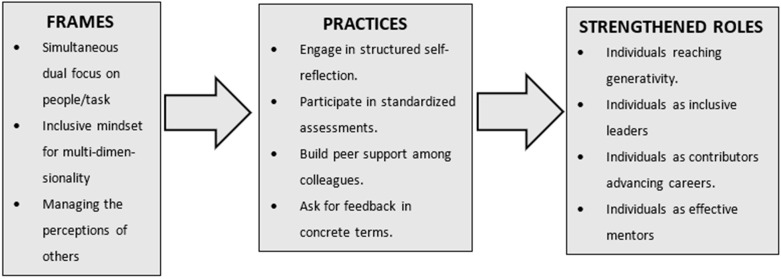
Frames, practices, and strengthened roles.

We challenge the myth that everyone who wants to be a mentor arrives prepared and ready to work [22]. We frame questions that mentors might use to deepen their self-awareness and promote their growth as leaders (see Figure 1). These frames are paying attention to people and tasks; working across multi-dimensional social identities; and managing the perceptions of others. The behavioral practices that encourage mentors to intensify their self-knowledge are engage in self-reflection; participate in standardized assessments; build a constellation of peer support; and ask for feedback.

### 4.1. Frame 1: People and Task

Kram [17] (1983) outlines two primary roles that mentors play: they act as providers of both psychosocial and career support. The mentoring literature clearly illustrates the importance of mentors’ skill at paying attention to both when supporting mentees. Another way to think about these dual roles are mentors’ ability to focus on both people (as they enact the psycho-social role) and task (as they enact the career role). This reframing of the lexicon connects the mentoring literature to leadership scholarship and reveals arguments for why mentors need to be developed as leaders who have the dual lens of people and task.

Between 1940 and 1986, sixty-five classification systems of leaders’ behaviors were proposed. In almost all of them, behaviors were broken into two categories: task-focused and people-focused [23]. This dichotomy was even present in the earlier trait scholarship, determined when Stogdill [24] revisited traits in 1970, placing them into six categories, one of which was task-related and another was social.

Over the years, scholars continued to build models that relied on the task-people paradigm [25,26,27,28] with a dual focus designated as optimal. Concurrently, other scholars sought to determine the impact that leaders’ behaviors had on team outcomes, such as perceived team effectiveness, productivity, and learning. While studies have produced somewhat mixed results, Burke et al. [29], through their meta-analyses of 231 studies between 1900 and 2004, built a leadership framework drawing from both task and people behaviors which explained a significant amount of variability in team outcomes.

In organizational practice, this dual focus on people and task translated into focusing on what gets done and how work gets done. For mentors, this duality is seen in the socio-emotional (people) functions and career (task). However, importantly, for mentors who seek to contribute and lead, developing their dual capacity will further their ability to develop strong relationships with individuals crucial to their success; lead teams more effectively [30]; and generally, increase their contributions to organizational goals [31].

While this capacity to enact both task and people focused behaviors has been well established, so is the perceived gendered nature of the behaviors, too [32,33,34,35,36,37]. Task-focused behaviors have long been conflated with masculinity, while people-focused behaviors have been conflated with femininity. Gender is one of many social identity dimensions that leaders in the current workplace context will negotiate. The ability to adeptly address issues of diversity are critical—which supports the essential importance of our second frame of development for mentors—multi-dimensionality.

### 4.2. Frame 2: Multi-Dimensionality

As global boundaries continue to be lowered and the professional landscapes change, there is an increasing need to work across various dimensions of social identities. Mentors’ relationships may be with people who are quite different from themselves. Clearly providing opportunities to strengthen mentors’ abilities to work across social identity differences is critical. While the importance of mentors’ ability to work with mentees of different social identities is well established [16,38,39,40,41], mentors as leaders also need this capacity strengthened. The framework of multi-dimensionality offers mentors support in strengthening their ability to work across dimensions of diversity in social identities, enhancing their capacity to build inclusive workplaces.

Drawing from legal scholar Kimberle Crenshaw’s theory of intersectionality [42], we stress multi-dimensionality as a core competence that leaders will need to be effective and must embody. Multi-dimensionality speaks to the reality that our human lives cannot be explained by examining a single personal category: our lives are too complex. The mentor who is paying attention to intersectionality understands that the experiences of a White, female, transgender mentee may be very different from the reality of an Asian, physically-challenged man.

For mentors in their roles as organizational leaders, the ability to work across a spectrum of dimensions of social identities is a must-have skill set. Chrobot-Mason and Leslie [43] suggest a number of beneficial outcomes that leaders with high levels of multicultural competence provide to an organization: they may be skilled at minimizing potential conflict that arises from increased diversity; they may show greater creativity; and they may explore alternative solutions more.

Understanding multi-dimensionality may also enable mentors to build inclusive work cultures. Blake-Beard et al. [44] argue that it is only when leaders “imagine the lives of their workforce” that they are able to build inclusive workplaces. For example, mentors may be more likely to expand policies and norms of ‘flexibility’ to make it relevant for people of different social identities: paid days off for religious holidays expand beyond legacy Christian holidays; and, time off for family goes beyond the conventional (and white) ‘nuclear family.’ A leader who is purposeful in creating an inclusive work culture will acknowledge different holidays and celebrations; ensure diverse team representation; and review organizational policies to identify potential biases. Chrobot-Mason and Leslie “predict that multicultural competency will be viewed as an important determinant of managerial success in the global workplace” [43].

### 4.3. Frame 3: Perception Management

Mentors operate in an environment where they not only need to do good work, but they increasingly need to manage how that work is perceived. While the #MeToo movement revealed many truths about gender, power, and organizational life, one critical lesson is the need to manage the perceptions of others [21]. Particularly relevant to mentors, at the height of #MeToo movement, was the immediate question: will men’s fears about being misinterpreted as they mentor or sponsor women result in a withdrawal from their co-ed roles? A *New York Times* article [45] summarized many of the ‘unintended consequences’ including men withdrawing from cross-gender mentoring roles or avoiding solo interactions [46]. Yet, women in a 2019 study [47] reported little change in their relationships with male mentors.

While women mentees have always had to manage how interactions with male superiors ‘look’ around the office, #MeToo powerfully revealed how superiors (bosses, mentors, sponsors) now need to do the same. Individuals, when mentoring or leading people who are in an organizationally subordinate group or in a socially less advantaged group (often women, people of color), need to do good work [48]. These mentors must also recognize how that work may be viewed through the lenses of others. One outcome of the increased diversity of people in organizations is the heterogeneity of perspective in how leadership is defined, how respect is shown, and how power is used, among other aspects of organizational life. With homogeneity, there is less potential for misinterpretation of motives.

In contrast, norms about respect, distance, and power vary across different social identity groups. For example, in their work on the mentoring experiences of Asian Americans, Chin and Kameoka [49] address the importance of acknowledging the impact of stereotypes and cultural values. They advise that “mentors should be aware of the cultural values that predominate in Asian American cultures—in particular, hierarchical collectivism and a high-context communication style” (p. 337). They found that when there is not an alignment between mentors and mentees’ cultural norms, mentoring relationships may be negatively impacted. Rehka and Ganesh [50] note that “India is also high in power distance, so the mentor is usually seen as a parent figure (associated with patriarchy and power hierarchy)” (p. 223). No longer can a person who is aligned with the dominant social identities of senior management behave in ways that their diverse constituents will correctly interpret and understand. While there are many explanations for perception errors with increased diversity, the errors of stereotyping, implicit leader prototypes, and unconscious bias have become more salient. Stereotypes regarding gender [35,48,51,52,53], and race/ethnicity [49,54,55], challenge White, masculine leader prototypes, and unconsciously impact how people of those identities are perceived and evaluated.

This is the environment in which mentors operate: in managing their careers and their reputations, mentors need to understand that their well-intentioned behaviors may be misinterpreted, and they need to be competent in employing strategies to minimize that misinterpretation [56]. In their mentoring roles, which is often highly visibility work [57], they need to be aware of the larger organizational context. How are their actions and relationships being interpreted by other players? What are the costs of different associations [58,59]? Mentors’ awareness of their surrounding context, knowledge of the unspoken organizational rules, and insights into possibly conflicting expectations are important for the mentors’ success.

## 5. Practices in a Mentor Developmental Framework

The following four behavioral practices operate in concert with one another to deepen mentors’ self-knowledge. The four are engaging in self-reflection, participating in standardized assessments, building a constellation of peer support, and asking for feedback. Engaging in these practices may lead mentors towards generativity. These practices will also enable mentors to be more successful in navigating the frames throughout their own careers and in their leadership roles. Finally, the practices enable mentors to experience and then role model many of the activities they may facilitate with their mentees.

### 5.1. Practice 1: Engage in Self-Reflection

Self-reflection involves the art and science of seeing ourselves, our emotions, and our actions with accuracy and clarity. Appling the findings from brain-research, contemporary writers have joined the chorus from centuries-old philosophers and religious thinkers to “Know Thyself.” Writers, such as Daniel Goleman [60] in his ground-breaking book, *Emotional Intelligence: Why It Can Matter More Than IQ,* can lead a mentor through the steps of identifying and naming “primary” emotions, such as anger, fear, and shame to enjoyment, love, and surprise (p. 289). Regardless of the writer, there is consistency in the perspective that a new practitioner of self-reflection requires a structured approach and commitment of time and energy.

The benefits of clear and accurate self-awareness through self-reflection are multiple. Mentors who can evaluate and manage their own emotions can aid others in doing the same [61]. Mentors who use emotional intelligence to manage themselves can also lead teams to do the same. Mentors who know how to regulate the range of their emotions become role models. Such self-regulation may come through different processes. Hamilton [62] argues that leadership development programs are one way to begin: program leaders should illustrate to participants the usefulness of articulating and journaling their range of emotions after they receive feedback. Writing responses in a journal makes an emotion concrete and encourages a mentor to return to and reflect upon their feelings. Porter [63] gives the practical advice to try a self-reflection journal for seven days.

To begin a practice of self-reflection and increased self-awareness, mentors need to decide on their primary goal(s) and commit to journaling for a specified time period [64]. Increased self-awareness is not a one-shot deal, but rather comes as a result of commitment to an on-going practice. The following three practices of assessment, peer support, and feedback should yield data that will increase mentors’ acute picture of themselves. Increased self-knowledge through self-reflection may be both the easiest and the hardest way to develop as a mentor, as it requires self-discipline and precious time.

### 5.2. Practice 2: Participate in Standardized Assessments

The use of assessment is one avenue that is available for the development of mentors. The term “assessment” refers to a wide variety of validated, standardized methods or tools that mentors can use to evaluate, measure, and document their current state and their readiness for growth. These frameworks are available in numerous designs, but all are intended to measure and elucidate an element of character, behavior, dispositions, and/or temperament [65]. There are a number of assessments that are widely used for the development of self-knowledge that is critical for mentors.

The self-knowledge gained from assessments is important for several reasons. Allen and Eby [66] suggest that mentors, in part, seek mentoring relationships with others to serve their own developmental needs. Participating in mentoring relationships is a manner of interacting that fulfills their desire to use their self-knowledge as a strength in supporting their development. Assessments provide mentors with an avenue to stay current with trends and practices that can be helpful to them in the management of others, and information gained from assessments may make mentors more competitive as organizational leaders. The use of assessments also provides mentors with a space for professional development to accommodate diversity [1].

The Birkman Method, for example, is a 298-question self-assessment tool: it describes an individual’s normal and under-stress communication style and behaviors; needs from others; and interests. Research [67] supports the benefits of the Birkman Method as an approach to facilitate growth in self-awareness and self-confidence. These aspects may be beneficial to the development and maintenance of an effective and productive mentoring relationship.

### 5.3. Practice 3: Build a Constellation of Peer Support

One of the practices that mentors can pursue is to build a constellation of support for themselves. In other words, the mentor needs mentoring. Peer mentoring has been defined as “a helping relationship in which two individuals of similar age and/or experience come together, either informally or through formal mentoring schemes, in the pursuit of fulfilling some combination of functions” [68]. Kroll [69] suggests that it could include multiple people. Within peer mentoring, learning occurs through dialogue and social interaction [70]. Peer mentoring is also distinctive due to the mutual mentoring practice of the collaborators. Each participant serves as both mentor and mentee, simultaneously receiving support and providing challenge.

Peer mentoring is important for the development of mentors for several reasons. These relationships provide a space for mentors to be vulnerable. They contradict the common myth that mentors “know everything” [71,72]. Being in a space where mentors may openly indicate that they do not have the answer without fear of being negatively perceived is rare. Peer mentoring also provides participants who may go on to mentor in the traditional framework with a forum to practice the skills that they want to share with their mentees. For example, vigorous interactions with their peers offer the opportunity to strengthen conflict management skills. Peer mentoring may also offer peers the opportunity to learn best practices in their industry or field. Finally, peer mentoring may provide a space for mentors to enhance their reputation.

There are several examples where the benefits of peer mentoring are apparent. In a study of peer mentoring in pediatrics trainees, the trainees reported that the peer mentoring was useful and helped them develop perceived competencies in supervision, leadership, decision-making, and clinical skills [73]. In another study, Danzi et al. [74] found that peer mentoring is perceived among trainees as enhancing their profession-wide competencies during training. This high level of satisfaction was evidenced by the majority of participants choosing to continue as peer mentors for multiple years.

### 5.4. Practice 4: Ask for Feedback

Feedback can be a powerful means for human learning. It ranges from casual comments made by colleagues to formal, yearly performance reviews. In feedback exchanges, there are two actors: the giver and receiver. For decades, management training programs focused on givers, extoling them to provide specific, timely comments to employees, allowing them to correct their behaviors quickly. However, through their consulting work and research, Stone and Heen [75] found that it is the receiver who is the critical player in learning and growing from feedback.

Feedback differentially impacts receivers. Stone and Heen [75] posited that receivers are hard wired for how they will respond to comments. Some people cringe, as they fear that in a few words their short-comings will be openly displayed. For others, there is a nonchalant shrugging of shoulders and a “so what?” attitude toward any and all feedback. Other researchers have also noted that receivers respond differently to feedback. Most notably, researchers [76,77,78] have found that social identities play a role in how receivers are given and how they digest comments. For example, Johnson and Helgeson [79] found that men and women respond to evaluative feedback differently: men’s view of themselves was relatively unaffected by the nature of the feedback, whereas women’s self-esteem improved with positive feedback and dropped after negative comments.

For mentors in the upper ranks of their organizations, feedback has likely been handed to them throughout their careers, most notably as 360-degree data in pre-packaged leadership assessments. However, actual feedback on performance becomes less frequent and less consistent the higher the manager’s position within the organization [80]. Most relevant for mentors is the research that “shows that people who solicit feedback—especially negative feedback—tend to receive higher evaluations than those who don’t” [75]. Upper-level mentors whose positions may inhibit others from giving them feedback need to be vigilant in asking for it. Stone and Heen argue that the best way to do that is to ask: What is one thing that I should do to improve my performance in X (a specific area)? Importantly, mentors who ask for feedback signal to others and remind themselves of the need to continuously develop through a potent mechanism feedback.

## 6. Discussion: Benefits and Challenges for Mentors and Organizations

### 6.1. Benefits for Mentors

Through their participation in the developmental framework, mentors reap benefits, including addressing their need for generativity, building their reputation as talent developers, and building their capacity to contribute and lead.

A predominant benefit noted in the literature is that mentors’ report gratification from their participation in mentoring. Across industries and disciplines, mentors share the pleasure and sense of giving back that they feel as a result of participating and building the next generation of leaders [81,82]. This observation suggests that acting as mentors benefit leaders by enabling them to choose generativity versus stagnation [83].

Second, mentors also spoke of their reputations being enhanced inside their organizations due to their work in supporting mentees [84]. In many leadership models, some aspect of talent development is included as an essential role. It may be described as “enable others to act” or “coach others’ potential” [85]. Mentoring becomes a powerful way to showcase the developmental strengths of the mentor, solidifying their reputations as leaders and opening up further opportunities to contribute.

Finally, building capacity in the three frames will enhance mentors own job performance and career advancement [66]. For each frame (the duality of people and task, multi-dimensionality, and perception management) that we identified, the driving factors facing organizations propel the need for leaders who have a deep understanding for not only their own success, but how to build a responsive work culture.

### 6.2. Challenges for Mentors

While there are benefits arising from developmental activities, there are also challenges. Having time in an overscheduled life may be the first and most difficult challenge. Additional ones include vulnerability and competition.

Receiving assistance makes the mentor vulnerable in several noteworthy ways. First, mentors will need to acknowledge that they do not know everything, and that they can learn and better themselves. There is a tendency to attribute one’s successes to oneself to preserve one’s positive self-image [86,87]: this is a cognitive barrier most people must overcome to be receptive to development activities. Mentors’ often senior positions in organizations compound this: they may view their promotions as signals that their skills, behaviors, and knowledge are already at levels worthy of being rewarded. Mentors also face the question of whether or not they are open to reaching beyond their comfort zone to consider new strategies, paradigms, and information, and practice new behaviors. There is a level of vulnerability that is required to acknowledge that one is not omnipotent and can, in fact, add to one’s repertoire by being open to new information. That vulnerability powerfully impacts the practices of self-reflection and using standardized assessments.

The practices of peer mentoring and feedback involve receiving information from someone else. Dynamics may impact both sides of the equation. On the asking side, the mentor may need to overcome concerns about how they may be misinterpreted: do they believe asking for feedback may be perceived as lacking confidence? On the giving side, mentors may resist giving feedback if they equate doing so as essentially training their competitors for increasingly limited promotional opportunities. Mentors may need to build reciprocity into their relationships: what can they gain and how can they support colleagues’ development?

### 6.3. Benefits for Organizations

While this paper has focused on developing mentors so they can lead, contribute, and mentor from strengthened positions, the organizations in which they are members also stand to gain. One organizational benefit is the increase in retention of their top talent due to the reciprocal benefits accrued in a mentoring relationship. In a meta-analysis, Ghosh and Reio [15] established that being in the role of mentor can increase job satisfaction, organizational commitment, and career success of the mentor themselves.

Given the highly competitive market for talent, mentoring can be an effective retention strategy. Johnson and Smith [10] argue that by building a “mentoring culture,” an organization will garner “better retention, more loyalty and commitment of employees, stronger succession planning, more organic mentoring and strengthening of resilient networks (p. 3).” The Society for Human Resource Management reported on a study that found that the number one reason employees left their jobs in 2018 was the lack of career development [88]. Mentor development could be a strong message to the contrary.

Reducing turnover provides significant financial benefits. U.S. businesses incurred the cost of one trillion dollars annually for voluntary turnover [89]. Included in that annual study, Gallup cited the Bureau of Labor Statistics’ report that the overall turnover rate in the U.S. in 2017 was 26.3%, and went on to conservatively estimate that the cost of replacing an employee is between one-half to two times their annual salary.

Over 70% of Fortune 500 companies reported having mentoring programs as an employee retention strategy and 25% of smaller companies did [90]. Given these numbers, and the number of admonitions about how mentoring programs are often conducted poorly, most likely there is great variability in programs across companies and industries. One key to ensuring a successful mentoring program may be in developing the mentors themselves.

## 7. Implications for Future Research

In this paper we identified a gap in the mentoring literature and constructed a conceptual model to fill that gap. The model is informed by our years of work with mentors in all types of organizations. We propose that a critical next step is to explore the impact of our model. As we practice in the field, we have anecdotally been told of positive outcomes by mentors in often unsolicited feedback: promotions, expanded responsibilities, successful DE&I efforts, recognitions as valued contributors. There has been a rigorous scholarly effort to determine the impact mentoring has had on the mentee [6,7,9], and on the mentor in their role as mentor [11,12,13].

We propose that research could affirm, challenge, and improve our conceptual model by examining the question: Has developing a mentor’s three frames through the use of the four practices enabled them to (see “Strengthened Roles”in Figure 1)

- Increase their own feelings of generativity? Does developing mentors result in their greater job and personal satisfaction and contributions to their organizations?
- Increase their ability to serve as an inclusive leader? In what ways does the organization benefit from leaders’ increased awareness of their diverse employees?
- Increase their ability to advance their careers? And how is the organization impacted: is there increased retention of senior individuals?
- Increase their effectiveness as a mentor in modeling the three frames?

We have briefly mentioned the dynamics that social identities may play in developing mentors. Further research is needed on how these frames and practices may be performed across social identities. For example, women may receive feedback to enact leadership behaviors that are conflated with masculinity. How does she work with a feedback-giver whose advice reflects their own social identities and not hers? In a 2018 study, 77% of mentors reported that they have relationships only with mentees of the same gender and race: clearly, additional research is critical on how to mentor across diverse identities [91].

A third consideration of future research is to delve into the importance of self-care. Mentors are not immune to the increased stresses in today’s environment, and fortifying their capacity to manage stress is crucial. Mentors are doing hard work, and wrestling with dynamics which generate their own stress. However, there’s another reason mentors need development in managing stress: it is in their roles as mentors. While mentors want to be empathetic as part of their work with their mentees, learning how to do so without absorbing the stresses of the mentee is essential.

## 8. Conclusions

In this paper we sought to fill a space that has not been sufficiently examined: how to develop mentors to build their capacities as mentors and, concurrently, to develop and sustain them as leaders. Focusing on this gap addresses mentors’ needs for generativity at a junction in their work lives when they can choose to grow while developing the future generation of leaders, or they can choose to stagnate and/or leave. Our frames and practices are powerful tools to support mentors on their continuing leadership journey.

A focus on the development and support of mentors is essential to benefit them, their mentees, and their organizations. A thoughtful understanding about how to create practices, policies, and spaces that nurture mentors mark a forward-thinking perspective that will be essential as we continue to see change across organizations, labor markets, and nations.

## Figures and Tables

**Table 1:** Kram’s roles of mentors (1985).

Function in Career Role	Function in Psychosocial Role
Helps me attain desirable positions	Is someone I can confide in
Uses their influence in the organization for my benefit	Provides support and encouragement
Uses their influence to support my advancement	Is someone I can trust
Suggests specific strategies for achieving career goals	Frequently socialize one on one outside the work setting
Gives me advice on how to attain recognition	Frequently get together informally after work by ourselves
Helps me learn about other parts of the organization	Serves as a role model for me
“Runs interference” for me	Represents who I want to be
Shields me from damaging contact with important people in the organization	Is someone I identify with
Protects me from those who are out to get me	Guides my personal development
Provides me with challenging assignments	Serves as a sounding board for me to develop and understand myself
Assigns me tasks that push me to develop new skills	Guides my professional development
Gives me tasks that require me to learn new skills	Accepts me as a competent professional
Helps me be more visible in the organization	Thinks highly of me
Creates opportunities for me to impress important people in the organization	Sees me as competent
Brings my accomplishments to the attention of important people in the organization	

## Data Availability

There were no datasets used.
